# Supplementary material for: Are carbon emissions trading and green financial instruments synergistic? -Comprehensive quantitative research based on content analysis
Source: PLoS One. 2024 Mar 7;19(3):e0298601. doi: 10.1371/journal.pone.0298601 (PMC10919601; doi:10.1371/journal.pone.0298601)
Supplement: S1 Data — (ZIP) [file pone.0298601.s001.zip › Data/地级市Z_F工作报告.zip!/╟α║ú/╬≈─■╩╨/└┤╫╘╣1⁄2╓┌║┼í╛┬φ┐╦╩2╛▌═°í┐_2008.docx]

各位代表：

现在，我代表市人民政府，向大会作工作报告，请予审议，并请市政协各位委员提出意见。

一、2007年政府工作回顾

2007年，在省委、省政府和市委的正确领导下，我们坚持以科学发展观为统领，紧紧围绕科学发展、保护生态、改善民生三大任务，与全市各族人民一道共同努力，全面完成了市十四届人大二次会议确定的各项任务，经济社会发展取得新的进展，呈现出六个显著特征：

————综合实力迈上新台阶。生产总值突破300亿元，达到343.1亿元，增长15.3%，占全省GDP的比重达到45.7%，比上年提高2个百分点；实现地方一般预算收入18.4亿元，增长30%，是近年来增幅最高的一年。

————工业发展再创新业绩。工业经济速度和效益协调增长，实现工业增加值147.9亿元，增长26.4%，对GDP的贡献率达到66.4%，带动GDP增长10.2个百分点；规模以上工业企业实现利润33亿元，增长24%。

————城乡面貌发生新变化。投资力度加大，结构不断优化，固定资产投资完成178.9亿元，增长26.9%，基础设施和生态环境明显改善，发展后劲进一步增强。

————社会民生得到新改善。年初确定的28项为民办实事项目、10项为农民办实事项目全部落实，使发展成果更多惠及民生。

————城乡居民收入实现新提高。城镇居民人均可支配收入突破万元大关，达到10636元，增长13.9%；农民人均纯收入3398元，增长15.1%。

————节能减排取得新成效。单位地区生产总值能耗下降2.5%，化学需氧量减少1100吨、二氧化硫排放量减少1700吨，主要污染物排放量出现近年来首次下降。

各位““代表””，2007年，是全市上下抢抓机遇、加快发展的一年，是奋力拼搏、扎实苦干的一年，是经济快速增长、民生显著改善、城市形象大幅提升的一年，也是为全面实现"十一五"规划奠定良好基础的一年。一年来，我们主要做了以下工作：

（一）扎实推进新农村建设，农业实现增产增效。紧紧围绕社会主义新农村建设，整合资金7.9亿元，加大对农村基础设施建设和特色农业发展的投入，改善了农村生产生活条件。新建农村公路604公里，改造村级道路1515公里。建成日光节能温室3015栋，完成6217户"一池三改"项目，建设各类养殖基地28个、牲畜暖棚3008栋。粮食、油料、蔬菜产量分别比上年增长10.1%、4.2%和2.7%，实现了农业增产增效。贩运育肥牛羊123.4万头只，生猪出栏46.2万头，畜牧业增加值达到8.5亿元，增长7.1%。龙头企业的带动作用明显增强，马铃薯、油料、蚕豆等"订单农业"面积扩大，带动农户15万余户。实施"阳光工程"，完成农村劳动力培训28.2万人次，转移就业32.5万人次。加大扶贫开发力度，在47个绝对贫困村实施了扶贫开发整村推进项目，解决了4.8万人的温饱问题。

（二）转变经济发展方式，工业化进程步伐加快。把工业经济作为转变发展方式、推动发展的主动力，围绕延伸产业链，培育壮大特色经济，加快工业项目建设，全市共有151个项目开工建设，维德铜业、青海明胶、鸿洲陶瓷等73个项目已建成投产，多晶硅、单晶硅、铜箔、百万吨复合肥等一批重大工业项目正在抓紧建设，以省内资源为依托，以新型材料为主体的高科技产业正在形成。突出发挥园区的引领作用，加强园区供电、道路、铁路、供排水、污水处理等基础设施建设，改善园区发展环境，提高土地资源利用效益，园区产业集聚能力明显提高，发展势头良好。园区全年完成工业增加值38.1亿元，增长68.1%,占全市工业增加值比重为25.7%，比上年提高8个百分点。

（三）发挥旅游业的带动作用，社会消费不断扩大。加强旅游基础设施建设，加快旅游产品开发，加大旅游资源营销力度，全力打造"中国夏都"品牌，实现了旅游业蓬勃发展。全年共接待国内外游客689万人次，增长37.1%。旅游业总收入突破30亿元，达到30.9亿元，增长42.3%，增幅比上年提高20.7个百分点。"浦宁之珠"观光塔、湟源石刻文化走廊、西路军纪念馆等景点建设进展顺利，新建和改造了一批宾馆、酒店及其他旅游配套基础设施项目，旅游接待服务能力不断增强。朝阳物流园区建设取得新进展，青藏高原小商品集散中心等市场开工建设，"万村千乡"市场工程项目加快实施。认真开展产品质量和食品安全专项整治行动，违法违规生产经营行为得到有效遏制，市场监管体系进一步健全，市场秩序逐步规范，居民消费环境明显改善，社会消费品零售总额达到138.9亿元，增长16.3%。

（四）加大城市建设投资力度，城市功能更加完善。坚持以规划为龙头，进一步加大城市基础设施及生态环境建设投入，建成了建国南路、朝阳东路等重点工程，周家泉片区综合开发、果洛路、大南山主骨架公路、儿童公园改造、湟水河湟中桥至宁湖段综合治理等项目进展顺利。全面启动海湖新区开发建设，完成了新区总体规划和景观等专项规划，五四西路延伸段等新区主干道工程、青海科技馆等大型公建项目开工建设，新区村民安置小区工程陆续开工。投资1.5亿元，完成28项小城建项目，是投资力度最大的一年，使居民出行环境得到明显改善。城市亮化、美化等工作取得新进展，城市面貌进一步改善。

（五）深入推进节能减排，生态建设成效明显。认真落实有关政策措施，着力推进节能减排工作。投资5.9亿元，在29户重点企业组织实施了节能降耗、节水及资源综合利用项目；建成一批以粉煤灰、煤矸石等工业废渣为原料的新型节能环保型企业，工业固体废弃物的综合利用率达到55.8%；落实14个化学需氧量减排项目、15个二氧化硫减排项目，关停了一批高耗能、高污染、低效益的小硅铁、小水泥、粘土砖厂等企业；开展维护群众健康环保专项行动，启动了新一轮煤烟型污染整治工作；建成西宁第二污水处理厂，城市污水处理能力进一步提高。生态建设加快推进，完成大南山绿化2万亩，建成开放湟水森林公园、鲁青公园和浦宁友好园，八一路等城市主要干道绿地景观改造顺利完成，积极推进城郊绿化、退耕还林、天然林保护等重点生态工程建设，城区栽培树木97.4万株，种植草花17万平方米，荒山造林7.8万亩，栽植沙棘林5万亩。创建国家园林城市、国家卫生城市取得新成效，荣获"省级环保模范城市"称号。

（六）不断扩大改革开放，发展活力明显增强。着力推进农村综合改革，优化设置乡镇机构，建立义务教育经费保障机制，理顺了"乡财县管乡用"的财政管理体制。推进城市管理体制改革，建立了新的管理、监督、考核运行机制，城区管理城市的主体地位得到强化，管理水平进一步提高。积极推进城市管理相对集中行政处罚权试点工作，组建了城市管理行政执法机构。加大公交体制改革，进行企业重组，公交资源得到优化配置，企业运行质量和服务水平明显改善。推进文化体制改革，发展活力得到增强。民营经济加快发展，私营企业达6600多户，实现销售收入113.7亿元，增长10%。加快对外开放步伐，强化招商引资的责任和措施，改进招商方式，招商引资的质量和水平上了新台阶，省外投资增长12.3%。扩大与国内外的交流与合作，与广州市缔结为友好城市，成功举办和承办了"郁金香节"、"青洽会"、"国际藏毯展览会"、"国际清真食品博览会"等大型活动，"夏都西宁"对外影响进一步扩大。

（七）注重统筹协调发展，社会事业全面进步。不断增加对社会事业的投入，促进了社会事业的健康快速发展。制定并实施《西宁市教育布局结构调整方案》，完成了城北小桥地区中小学资源整合，新建沈那中学，对20所中小学危旧房进行了改造。加强职业教育，实施了世纪职业学校扩建项目，加强校企合作，提高职业教育专业建设水平，大力开展各类职业培训，劳动者技能不断提高，创业就业能力得到增强。进一步加强科技工作，实施科技项目44项，科技创新能力不断提高。公共卫生体系建设得到加强，疾病预防控制能力明显提高。城市社区卫生服务进一步规范，社区卫生服务人口覆盖率达到了95%，新型城市卫生服务体系正在形成。农村卫生基础设施建设步伐加快，建成农村卫生室425所。开工建设了流浪少年儿童救助保护中心。积极发展文化事业，建成了数字电视公共信息服务平台，努力繁荣文艺创作，群众性文化体育活动蓬勃开展，竞技体育取得新的突破。实施计划生育"奖励扶助"、"少生快富"制度，人口自然增长率为7.6&permil;。

（八）着力保障和改善民生，社会建设和谐有序。深入开展就业再就业援助活动，努力拓展就业再就业渠道，实现城镇新增就业2.1万余人，下岗失业人员再就业1.5万余人，安置零就业家庭成员就业2400人（次），实现了零就业家庭的动态清零目标。关心贫困群众生活，对89万人次城镇低保对象发放低保金；建立了农村最低生活保障制度，将8.4万人纳入保障范围，11万农村受灾群众的基本生活得到妥善安置；免除了农村和城镇低保家庭义务教育阶段所有学生的学杂费；新型农村合作医疗覆盖面进一步扩大，农民参合率达到97%，保障标准进一步提高；积极推进城镇居民基本医疗保险，参保人数达21.7万人，对7.5万城镇低保对象实行了医疗救助。高度重视社会救助工作，提高农村最低生活保障标准和医疗救助标准，增加了环卫等公益岗位人员的工资标准。建立了以廉租房、经济适用房为重点的城镇住房保障体系，1546户特困低保家庭列为住房救助对象，10万平方米经济适用房、240套廉租房建设进展顺利。关心企业离退休人员和老年人生活，为9万余名企业退休人员与1457名企业离休人员增加了养老金和生活补贴，对7.8万名65岁以上老年人在乘坐公交车等方面提供了免费服务。同时针对物价过快上涨，采取积极措施，平抑物价，为困难群体发放了临时生活补贴。

（九）加强民主法制建设，安定团结局面进一步巩固。加强民主法制建设，扎实推进依法治市，自觉接受人大及其常委会的法律监督和政协的民主监督，共办理市人大““代表””建议110件、市政协委员提案207件。加强政府自身建设，不断优化政务环境、提高行政效能，被评为全国政务公开工作先进单位。对现行的行政法规规章进行了清理，宣布失效、废止11件。加大人民来信来访工作力度，有效化解社会矛盾，信访量明显下降。认真落实党的民族宗教政策，扎实推进"平安西宁"建设，民族团结、社会稳定的政治局面得到巩固和发展。双拥工作取得新进展，连续五次荣获"全国双拥模范城"称号。审计、监察、工商、统计、质监、药监等工作取得新进步，妇女儿童、残疾人等事业有了新发展。

各位““代表””，2007年全市经济社会发展开创了新的局面，各项主要指标创历史最好水平，这些成绩的取得，是省委、省政府和市委正确领导的结果，是市人大、市政协积极支持的结果，是全市人民共同奋斗的结果。在此，我““代表””市人民政府，向各位人大““代表””、政协委员，向各民主党派、工商联、无党派人士和人民团体表示衷心的感谢！向奋斗在全市各条战线的工人、农民、知识分子、干部职工和各行各业各界的建设者和劳动者，向中央驻宁单位、省直各部门、人民解放军驻宁部队指战员、武警官兵和公安干警致以崇高的敬意！向所有关心、支持西宁建设和发展的各界人士，表示诚挚的谢意！

在总结成绩的同时，我们清醒地看到，"市不强、民不富、底子薄、基础差、积累少"仍然是我们的基本市情，经济社会发展中还存在许多困难和问题，主要体现在：农业基础依然薄弱，产业化程度低，农业增效、农民增收的难度大；工业经济总量小，结构单一，企业自主创新能力不足，资源节约、节能减排压力大；现代服务业发展滞后，服务体系不完善；财力不足，公共服务能力弱，改善民生的任务十分艰巨；物价持续上涨，群众生活特别是低收入群众的生活受到一定影响。同时，政府自身建设有待进一步加强，政府服务水平有待进一步提高，发展环境需要进一步改善。对于以上这些问题，我们要高度重视，切实采取有效措施，认真加以解决。

二、2008年工作的总体要求和主要任务

今年是全面贯彻党的十七大精神的开局之年，是落实省第十一次党代会精神和实施"十一五"规划的关键之年，也是加快西宁经济社会发展的重要之年。

根据市委十二届四次全体会议的安排部署，今年工作的总体要求是：全面贯彻党的十七大精神，高举中国特色社会主义伟大旗帜，以邓小平理论和"三个““代表””"重要思想为指导，深入贯彻落实科学发展观，按照省第十一次党代会和省委十一届三次全体会议对全省工作的总体部署，以做大做强西宁为目标，以建设东部综合经济区为己任，以项目建设和招商引资为关键，把科学发展作为第一要务，保护生态作为重要责任，改善民生作为当务之急，突出园区经济的引领作用和县域经济的支撑作用，积极推进改革开放和自主创新，加快优化经济结构、提高经济发展质量，加快节能减排、切实保护生态环境，加快改善民生、力促社会和谐，不断开创经济社会又好又快发展新局面，为把西宁建设成为更加繁荣、更加美丽、更加宜居的青藏高原现代化中心城市而努力奋斗。

主要奋斗目标是：

————经济较快增长，发展质量进一步提高。地区生产总值增长14%，地方一般预算收入增长17%，单位生产总值能耗下降3%，化学需氧量和二氧化硫排放量分别控制在3.1万吨和7.4万吨以内；

————固定资产投资平稳增长，社会消费进一步加快。固定资产投资增长17%，社会消费品零售总额增长14%；

————人民生活继续改善，价格涨幅得到合理控制。城镇居民人均可支配收入增长9%，农民人均纯收入增长10%，城镇登记失业率控制在4.5%以内，人口自然增长率控制在8&permil;以内，居民消费价格总水平涨幅控制在略低于去年实际涨幅。

今年，国家继续加大宏观调控，实施稳健的财政政策和从紧的货币政策，经济运行的环境进一步趋紧，对经济社会发展提出了更新、更高的要求，我们面临的困难增多，压力增大。站在新的发展起点上，为全面实现经济社会发展的奋斗目标，我们将坚持不懈地把发展作为西宁最重要、最紧迫、最重大的任务，不断增强工作的预见性，把握工作的主动权，坚持在发展中顺应调控，在调控中谋求发展，坚持科学发展，突出重点，统筹兼顾，着重抓好以下八个方面的工作：

（一）突出农业增效农民增收，扎实推进新农村建设

按照形成城乡经济社会发展一体化新格局的要求，坚持多予少取放活的方针，巩固和完善支农惠农强农政策，加强农畜产品基地建设和农业基础设施建设，促进农业稳步发展、农民持续增收，确保为农民办实事项目落到实处。

加快发展现代特色农业。按照服务城市、富裕农民的要求，大力发展现代农业，提高农业综合生产能力。着力推进蔬菜、油菜、马铃薯、蚕豆等优势农产品基地建设，着力推进花卉、食用菌、沙棘、中藏药材和优质饲草等特色种植基地建设，着力推进生猪、肉牛、奶牛、育肥羊及蛋禽基地建设。新建日光节能温室3500栋，牲畜暖棚3000栋，新建标准化奶源、肉牛和生猪基地各10个。蔬菜产量达到45万吨，自给率达到40%；乳品产量达到8万吨，自给率达到60%；肉类产量达到10万吨，牛羊肉自给率达到80%，猪肉自给率达到60%，畜牧业增加值占农业增加值的比重达到56%。进一步加强动物疫病的监测和防控，建立健全农产品质量检测体系，提高农畜产品质量和安全。

提升农业产业化经营水平。加大政策扶持力度，完善产业化经营模式，重点培育10户龙头企业，发挥龙头企业带动结构调整、促进农民增收的作用，创出一批优质、绿色的农畜产品品牌，推进农畜产品转化增值，形成具有特色优势的产业集群。重点扶持15家农业专业合作社，壮大农村经纪人队伍，提高农民的组织化程度。加强农村市场体系建设，完善农畜产品销售服务体系。规范农畜产品交易，积极开拓省外市场，努力增加农民收入。

加强农村基础设施建设。继续推进以农田水利、设施农业、乡村道路等为重点的农村基础设施建设。投资9600万元，继续实施县乡公路改造工程，建设里程达到178公里。加快盘道水库灌区一期工程建设进度。投资3000万元，解决5万人、4.5万头只牲畜的饮水安全问题。加大农村新能源建设力度，增加1.1万户沼气和5000户太阳能用户。进一步完善160个新村建设规划，加强农村教育、卫生、文化、体育等设施建设，继续整治村容村貌，改善农村人居环境。

大力发展劳务经济。有针对性地开展多渠道、多层次、多形式的劳动技能培训，组织实施"阳光工程"和"新型农民科技培训工程"，特别要加大初、高中毕业回乡青年的培训力度，培训农村劳动力2.2万人，着力培养一批具有较高文化素质和一定专业技能的生产能手、能工巧匠、经营能人和科技示范户，加快农村富余劳动力向非农产业转移就业，稳步扩大劳务输出规模，延长在外务工时间，农村劳动力实现转移就业30万人次，其中新增长期相对固定农民工2万人。

继续实施整村推进项目和小型易地扶贫项目，坚持扶贫开发与新农村建设、现代农牧业、劳务经济相结合，通过产业扶贫、教育扶贫等方式，改善农民生产生活条件，解决50个村7800户、3.5万贫困人口的温饱问题。

（二）壮大和提升特色产业，加速新型工业化进程

把转变经济发展方式作为关系全局的重大战略任务，立足西宁实际，调整产业结构，推进资源转换，促进产业优化升级，努力实现工业跨越式发展。

着力转变发展方式。大力发展循环经济，把发展循环经济、产业结构优化升级、转变发展方式紧密结合起来，积极促进产业之间的循环式组合、企业之间的循环式生产，着眼于资源综合利用和产业链延伸，推进建设一批优势资源与强势企业、优势资本、先进技术相结合的循环产业项目，提高资源的综合开发、循环利用水平。不断提高各生产要素配置效率，着力培育一批主业突出的大公司、大集团，在扩大工业规模中，优化工业发展的结构，提升工业发展的效益，增强工业对经济社会发展的支撑能力。抓住国家批准西宁经济技术开发区列为第二批循环经济试点园区的机遇，抓紧编制实施方案，推动园区发展上新的台阶。

壮大提升优势特色产业。大力培育发展硅材料、太阳能等新兴产业，重点抓好多晶硅、单晶硅以及铜箔、铝箔等项目，尽早形成生产规模，发挥效益。加快有色金属、复合肥、镍合金等项目建设进度，力争一批骨干项目早日投产。抓好沙棘、马铃薯淀粉、菊芋粉、伊斯兰民族服饰等扩产项目建设，积极做好中藏药企业的整合重组，发挥产业优势，做大企业规模。抓好机织藏毯、绒纺、羊绒分梳等项目建设，加快西宁国际性藏毯生产集散地建设步伐。加快大华工业园规划和基础设施建设，启动百万吨硅系列项目。加快传统产业改造，坚持用现代产业链条和产业集群的理念，整合和提升机械、建材等传统产业，促其生产上规模、质量上档次，做大做强产业。

提高企业自主创新能力。认真实施《全民科学素质行动计划纲要》，不断提高公民的科学素质，增强创新能力。继续开展以企业为主体、市场为导向、产学研相结合的技术创新体系建设，坚持以产品创新为核心，以创新、转化、产业化为主线，着力推进特色优势产业等重点领域的产品与技术创新，开发一批具有自主知识产权、技术水平先进、市场竞争力强的新产品，逐步提升科技对经济发展方式转变、和谐社会建设的支撑引领作用。

增强县域工业的支撑作用。按照城乡产业分工协作的要求，以强化农牧业基础地位为先导，以县域工业发展为重点，进一步明确发展重点，优化产业布局，大力发展特色产业，培育产业集群，促进县域经济快速发展。一是坚持多种产业并举、协调推进，落实发展措施，不断增强县域经济实力。二是围绕现有大企业和工业园区，加快发展协作配套的特色中小企业，不断延伸产业链，提高工业在县域经济中的比重。三是围绕区域特色和资源优势，积极发展矿产资源开发利用、农畜产品加工等行业企业，形成产业优势互补、协调发展的县域经济新格局。

加强工业运行协调服务。积极协调解决企业运行中存在的困难和问题，加大对在建项目的协调，促使项目尽快建成；对已建成但尚未投产的项目要跟踪服务，促使尽快投入运行，形成新的经济增长点，确保工业增加值增长20%以上。

（三）加快发展高原特色旅游业，提升第三产业的层次和水平

围绕建设旅游名省的总目标，精心打造"中国夏都"旅游圈，大力发展会议经济，积极培育新的经济增长点。

着力推进旅游业发展。围绕民族文化、高原自然人文景观等资源，进一步完善旅游业发展规划，制定操作性强、以项目为支撑的实施方案，加快旅游资源的开发和保护，推进旅游业快速发展，实现旅游业总收入增长30%以上。不断完善和丰富塔尔寺、中国藏医药文化博物馆等知名旅游景点的设施和内涵，加快大通鹞子沟、湟源明清老街等景点建设，提升旅游文化品位，不断发挥西宁旅游业的辐射作用。继续完善旅游配套基础设施，加快一批宾馆酒店开发建设，加强公路等交通体系以及餐饮、娱乐等相关配套建设；抓好地方特色旅游商品开发生产、市内大型旅游购物场所（市场）等项目，挖掘西宁历史文化资源，丰富旅游文化内涵，不断满足旅游业发展的需要。进一步做好宣传推介工作，扩大对外影响，加强与周边地区和主要客源地的合作，形成全方位的旅游产业开放合作体系。加大监管力度，做好景区景点环境保护工作。规范旅游市场秩序，加强旅游行业管理，深入推进诚信旅游和行风建设，不断提高旅游业服务水平。

大力发展会议经济。充分发挥"中国夏都"品牌作用，积极开发会展资源，制定会议经济发展规划，加强会议场馆等硬件建设，加快相应配套服务设施、中介机构和相关产业的发展，搞好会议人才培训，做好对外宣传和营销，积极争取申办、组织各种国际性、全国性会议，把西宁打造成全国夏季会议中心。进一步创新办会思路和方式，继续承办和协办好"青洽会"、"郁金香节"、"国际藏毯展览会"、"环湖赛"、"国际清真食品博览会"等赛事及会展活动，走出一条会议经济带动发展的路子。

推进现代服务业发展。大力发展现代流通业，加快推进朝阳综合物流园区建设，重点做好铁路物流中心、保税物流中心等项目建设，把朝阳物流园区建成联系全国、服务全省、辐射周边的重要物流集散地。按照功能齐全、交易规范的要求，加强农贸市场建设，完成7个市场改造工程。加快商业南巷步行街、中下南关民族用品一条街等重要商务街区的改造，提升市场的档次和品位。整合王家庄、东部综合批发等市场资源，建成全省最大的小商品集散中心。发展一批大型专业市场，大力发展连锁经营、特许经营等新型消费业态。加快农村消费市场建设，鼓励大型连锁企业把市场网络延伸到农村，实行商品统一配送、统一销售，促进农村消费平稳增长。积极发展信息、金融、保险、证券、中介等新兴服务业，促进服务业上水平、上规模。

（四）加快城市建设步伐，完善城市综合服务功能

深入实施"扩市提位"战略，优先支持海湖新区建设，加快老城区改造，不断完善城市基础设施，增强城市综合服务能力，促进城区建设协调发展。

突出规划的龙头作用。树立"以人为本、以环境为中心、可持续发展"的规划理念，依法加强规划管理，坚决维护城市总体规划的权威性。围绕"西陲重镇、高原古城、区域中心、中国夏都"搞好规划建设，挖掘、利用好悠久历史文化遗产，赋予城市新的时代内涵，保护和张扬城市个性，杜绝和防止趋同，使传统与现代、自然与人文有机结合起来，彰显西宁的美丽，不断提高城市规划、建设和管理水平，加快编制和完善土地利用总体规划、专项规划、分区规划和重点建设区域的详细规划，注重整体性、协调性、功能性和示范性，精心设计城市重点区段整体景观和标志性建筑，不断提升城市规划建设的品位。

强化城市功能的提升改造。围绕"扩大城市规模、完善城市功能、优化城市环境、提高城市品位、增强城市吸引力和辐射力"的建设思路，突出中心城区载体功能，营造城市特色。按照"一街一景"的要求，突出品位和特色，完成东西大街、南北大街、新宁路、黄河路、建国路、祁连路等"两街四路"景观综合改造，在整个改造建设过程中，注意实施好各类管线"入地"工程。全面实施19个片区城市景观改造工程，切实改善城市面貌，提高人居环境质量。投资1亿元，完成28个小城建项目，继续抓好重点区域和主要街道的灯饰亮化工程，不断提升城市品位。加强城市用地管理，坚持土地整片开发、整体出让，提高土地使用效益。加大土地收购与储备力度，增加土地储备规模，为城市建设提供基础条件和保障。

推进城区建设协调发展。按照功能明确、风格突出、相互协调、理念先进的要求，构建城区发展的新格局。城市东部，围绕打造具有民族特色、商贸繁荣的现代化城区，加快危旧房改造步伐，重点对周家泉片区、建国南路片区、火车站片区等进行综合开发建设。城市中部，围绕建设功能齐全、三产发达的现代化城市的核心区，立足于提升城市品位，拓展发展空间。重点做好城南新区和城中区合并的总体规划和分区规划，抓好城南温泉休闲度假中心、高原体育公园等项目，实现优势互补，协同发展。城市西部，围绕建设人居环境优美、配套设施齐全的现代化生态新城区的目标，重点加快海湖新区建设，年内基本完成新区主骨架道路的建设和火烧沟生态景观治理；青海大剧院、青海科技馆等标志性建筑建设要取得实质性进展。城市北部，围绕建设现代化综合物流和都市农业的城区，重点加快朝阳物流园区建设，完善配套基础设施，结合青藏铁路二线建设，加快北山危岩体综合整治工程步伐，实施地质灾害区居民的搬迁、土地整理及北山旅游综合开发项目，做好对西格线改造、丹拉国道西过境线等省重点工程涉及的拆迁、征地及服务工作。

三县城镇建设要突出重点、突出特色，围绕发展定位，从县城的整体功能和布局出发，统筹考虑规划，合理安排基础设施建设。同时，以三县县城为中心，带动周边中心城镇及小城镇的协调发展，形成经济分工有序、功能优势互补、空间布局合理、整体结构协调的城镇体系，加快城镇化进程。

大力改善生态环境。着眼于建设山清水秀、生态文明的高原宜居城市，继续加快大南山生态绿色屏障工程建设，新增绿化面积2万亩，全面完成一期48平方公里绿化任务，建成九眼泉生态观光体验公园。大力实施城市园林绿地建设，不断提升绿化水平，新建8处街头游园、改造单位庭院绿地20处。充分利用城市闲置土地建设临时绿地，不断扩大绿地面积。加快西塔高速公路和平西高速公路西宁段两侧的景观林带建设。扎实推进天然林保护工程、"三北"防护林建设工程，完成人工造林2.2万亩，种植沙棘林5万亩。加强水源地保护和水污染治理，积极推进城区及三县河道防洪世行治理项目。抓好湟水河等重点流域的污染防治工作。加强生态环境保护宣传，广泛开展绿色学校、绿色社区、环境友好企业创建活动。

提高城市管理水平。在巩固优秀旅游城市成果的基础上，进一步加大工作力度，切实抓好薄弱环节，加快创建国家卫生城市、国家园林城市和国家环保模范城市。坚持"建管同步、建管并重"，认真落实市容环境卫生责任制，整合社会资源，加大环境综合整治力度，解决影响市容市貌的突出问题。进一步创新城市管理、监督、考核机制，提高城市精细化管理水平。加强城市交通秩序和运输管理，不断改善城市交通环境状况。强化城市公共基础设施管护工作，依法严厉打击破坏城市公共设施的违法行为。

（五）加大节能减排工作力度，不断增强可持续发展能力

把节能减排作为优化经济结构的重要抓手，不断完善制度，落实责任，强化措施，在降低能耗、减少污染、提高效益上下功夫，实现经济发展和生态环境保护相协调。

一是建立科学、完整、统一的节能减排统计、监测和考核体系，把能耗降低和污染减排完成情况作为政府领导干部综合考核评价和重点耗能企业负责人业绩考核的重要内容，实行严格的问责制。把今年作为"节能减排攻坚年"，分解落实节能减排工作任务，强化节能减排的目标责任，采取更加有力的措施，推动节能减排取得明显成效。认真组织实施全国第一次污染源普查工作。

二是突出抓好重点行业、重点企业的节能减排工作，对高耗能、高排放重点企业要制定具体实施节能减排工程时间表，加大重点节能减排工程的实施力度，大力推广应用新技术、新工艺、新设备，实施一批效益显著的重点节能降耗和污染减排项目，努力降低能耗、水耗和污染物排放，确保重点企业污染物排放达标率达到85%以上。

三是对国家明令禁止、要求淘汰的落后生产能力摸清底数，积极争取国家和省上专项支持，加大对小钢铁、小水泥、小硅铁等高耗能、高污染、低效率企业关停并转的力度，加强对新上项目的用能、污染物排放量的审核工作，严把高耗能、高排放准入关。

四是加强建设领域节能减排工作力度。重点抓好新建民用建筑执行强制标准工作，开展城镇既有建筑节能和热计量改造工作，进行机关办公楼和大型公共建筑能效监测试点，大力推动太阳能等可再生能源在建筑中的规模化应用，推行城镇集中供热计量收费改革试点。开工建设第一污水处理厂二期工程，继续实施城镇供排水管网改造工程。

五是下大气力抓好煤烟型污染整治工作，消除主城区冒黑烟现象，改善城市大气环境。进一步组织实施环保专项行动，着力解决影响群众切身利益的环境问题。

（六）努力改善民生，推进和谐社会建设

把解决民生问题摆在更加突出的位置，加快推进以改善民生为重点的社会事业建设。落实好确定的15大类60项为民办实事项目，着力解决教育、医疗、就业、社会保障等群众最急需的民生问题，确保学有所教、劳有所得、老有所养、病有所医、住有所居，促进社会和谐。

优先发展教育事业。全面贯彻党的教育方针，坚持育人为本、德育为先，实施素质教育，提高教育现代化水平，培养德智体美全面发展的社会主义建设者和接班人，办好人民满意的教育。认真组织实施教育布局调整方案，整合教育优势资源，启动湟川中学新校区建设等项目，不断满足广大市民的教育需求。加大投资力度，完成6所城区中小学改造和40所农村危旧校舍改造工程。从秋季起，全部免除城市义务教育阶段学生杂费，提高农村义务教育经费保障标准，实现城乡统一的免费义务教育。进一步规范教育收费，健全学生资助制度，保障经济困难家庭和进城务工人员子女平等接受义务教育。更新教育观念，深化教学内容方式、考试制度、质量评价制度等改革，减轻中小学生课业负担，提高学生综合素质。加强教师队伍建设，重点提高农村教师整体素质。大力发展职业教育，抓好市第一职业学校迁建项目，以专业建设为核心，提高职业教育基础能力，加快培养技能型和实用型人才步伐。

大力推进就业工作。实施积极的就业政策，加强政府引导，千方百计为劳动者创造就业机会、提供就业岗位，使所有有劳动能力和就业愿望的劳动者尽早实现就业，使更多的劳动者成为创业者。紧紧围绕产业发展的用工需求，坚持政府培训、企业培训和职业技术培训相结合，加快高技能人才和适用人才培养，提高城镇新增劳动力的就业和创业能力。培训城乡各类劳动力2.5万人，新增城镇就业2.3万人，自主创业带动就业5000人。积极完善面向所有困难群众的就业援助制度，及时帮助"4045"人员实现就业，对零就业家庭实行动态管理，出现一户，解决一户。建立和完善城乡统一的劳动力市场，为劳动者提供就业指导、职业介绍等免费服务，改善就业环境。认真落实《劳动合同法》，加大劳动保障执法力度，维护劳动者的合法权益，构建和谐劳动关系。

健全社会保障体系。以社会保险、社会救助、社会福利为基础，以基本养老、基本医疗、最低生活保障为重点，以慈善事业、商业保险为补充，加快完善社会保障体系。继续做好社会保险扩面和基金征缴工作，重点做好各类非公有制经济从业人员、城镇灵活就业人员、个体工商户和农民工参加社会保险工作，保险扩面2.8万人。完善城乡居民最低生活保障制度，规范程序，加强动态管理。适当提高城市低保对象补助水平，关心低保边缘家庭生活，逐步改善生活条件。不断完善基本养老保险制度，提高企业离退休职工养老金标准，促进城镇职工基本养老保险制度规范化。全面推进城镇职工、城镇居民基本医疗保险和新型农村合作医疗制度建设，提高城市医疗救助和农村新型合作医疗政府补助标准，使基本医疗保险制度覆盖城乡全体居民。加快社会福利机构建设，完成市社会福利院迁建项目。健全社会救助体系，加强防灾救灾工作，做好优抚安置，发展残疾人事业。

完善住房保障制度。坚持把改善居民居住条件作为城市住房改革和房地产业发展的根本目的，特别把解决城市低收入家庭住房困难作为政府公共服务的一项重要职责，建立健全以实行货币补贴和实物配租相结合的廉租住房保障制度，对城镇住房困难的最低生活保障家庭应保尽保，积极构建多层次住房保障体系，完善住房供应结构，建立中低价位、中小套型商品住房供应机制，加快普通居民住宅建设，推进房地产业健康发展。在完成10万平方米经济适用房、240套廉租房建设的基础上，新开工建设廉租房1350套、经济适用房10万平方米、国有破产企业住宅区改造10万平方米以上。

强化公共卫生服务。坚持公共医疗卫生的公益性质，强化政府责任和投入，鼓励社会参与，加快建立有利于人民群众及时就医、安全用药、合理负担的基本医疗卫生制度。加强城市医疗卫生服务体系建设，打造城市居民步行15分钟就医的社区卫生服务圈。开工建设市第三人民医院门诊急救综合楼。进一步健全农村三级医疗卫生网络，新建标准化村卫生室456所。强化医疗卫生监管，加快卫生人才培养，狠抓医德医风建设，为群众提供安全、有效、方便、价廉的医疗卫生服务。抓好重大传染病、地方病的预防控制，提高突发公共卫生事件应急处置能力。继续稳定和完善现行生育政策，实施"奖励扶助"和"少生快富"制度，从严控制人口增长，提高出生人口素质。

积极建设和谐文化。加快基层文化建设，做好文物及非物质文化遗产的保护和开发，深入挖掘地方文化资源，激发各方面的文化创造力。组织实施我市"五个一"文化工程，重点打造一台““代表””西宁文化特色的剧目。完成30个村广播电视"村村通"工程，建立10个社区流动图书馆，不断丰富群众的文化生活。完善扶持公益性文化事业、发展文化产业、鼓励文化创新措施，繁荣文化事业。加强社区和农村体育设施建设，构建面向大众的群众性健身服务体系，提高群众体育活动普及程度和城乡居民健身水平。

扎实推进社区建设。按照建设管理有序、服务完善、文明祥和新型社区的要求，加快推进基层社会管理体制改革和创新，加大社区建设投入，改善社区工作环境，探索建立社区工作人员工资增长机制，实施好社区再就业服务中心等基础设施建设项目，进一步发挥社区自治组织的功能和作用。强化社区服务措施，拓展社区发展空间，完善社区家政、物业管理、体育健身、医疗康复、文化娱乐、托儿养老等综合服务。做好农村社区建设工作，增强农村社区服务功能。

(七)深化改革扩大开放，增强发展的活力和动力

按照构建充满活力、富有效率、更加开放、有利于科学发展体制机制的要求，加快重点领域和关键环节改革步伐。继续实施开放带动战略，着力完善内外联动、互利共赢的开放型经济体系。

加快行政机构改革。按照省、市行政管理体制改革的要求，调整完善部门职责权限，健全部门之间协调配合机制。健全政府职责体系，完善公共服务体系，强化社会管理和公共服务。进一步推进政府职能转变，继续清理、取消和下放行政审批项目，提高行政效能。认真落实《政府信息公开条例》，加快电子政务建设，推行政务公开。加强应急管理体系建设，提高危机管理和处置能力。稳步推进相对集中行政处罚权改革试点工作，建立起权责一致、运行协调、执法高效、监督有力的城市管理新机制。

推进事业单位改革。按照切实理顺管理体制、不断创新运行机制的要求，坚持机制灵活、渠道畅通的原则，稳步推进文化、卫生、科技等事业单位改革，创造有利于出成果、出人才、出效益的体制机制环境，进一步完善公益性事业单位公共服务职能，加快经营性事业单位社会化、市场化发展步伐，为社会事业发展注入新的生机和活力。

深化农村综合改革。继续以转变政府职能为核心，加强基层政府社会管理和提供公共服务的能力，形成精干高效的农村基层行政管理体制和机制。按照依法自愿有偿原则，积极探索土地经营权流转办法，发展适度规模经营；巩固试点工作成果，逐步建立运转协调、廉洁高效的基层行政管理体制；责任明确、财政分级负担、经费稳定增长的义务教育经费保障机制；县乡财政收入分配和支出结构合理、支农资金稳定增长机制。加大控制和化解乡村债务力度，加强对农民减负工作的监督管理，防止农民负担反弹。

抓好财政和投融资改革。围绕推进基本公共服务均等化，深化预算制度改革，加快公共财政体系建设。加强财政管理和税收征管，确保财政收入持续增长。深化支出改革，优化支出结构，努力满足重点支出需要。做大做强融资平台，推进融资方式创新，积极争取国债资金、专项资金和其他资金，鼓励和引导重点企业直接融资，进一步改善融资结构，充分调动各类投资主体的积极性，让沉淀的民间资金变成活跃的创业资本。完善城市建设偿债机制，防范政府债务风险。强化经营城市的理念，加大整合城市资源的力度，推进资源变资产、资产变资金、资金再投入的良性循环机制的建立，增强城市自我发展能力。

进一步扩大对外开放。抓住国家实施"万商西进"工程的机遇，进一步加大招商引资力度，创新招商引资方式。适应国家宏观调控的形势，把握投资重点，选准投资方向，编制一批好项目，充实完善项目库；对已有的项目要调整、完善和充实，提高项目工作深度；对所有项目，坚持分类指导，在加大与国家和省有关部门衔接汇报、争取支持的同时，充分利用各种平台，多领域、多渠道开展招商选商，特别要加强与国内外大企业、大公司对接，重点在引进一批能够带动产业链延伸、带动循环发展、带动发展方式转变的龙头产业项目上下功夫。加强对招商引资项目的审核管理，完善项目环保审核制度和程序，提高门槛，绝不引进高污染、低效益的项目，不断提高招商引资的质量。切实提高对项目的服务水平，对招商项目，无论是谁引进的，各地区、各部门都要结合职能分工，从政策落实、协调服务、合同签订、企业注册、项目建设等方面，全力做好工作，促进项目实施，确保全年招商引资到位资金增长10%以上。加强区域间经济联合和协作，巩固与友好城市的协作关系，拓展和加强与其他城市的交流与合作，建立多层次、多领域、多形式的对外交流与合作新格局。进一步加强环境建设，不断创新体制机制，强化协调服务，提高办事效率，适应发展需要。调整优化外贸主体结构、出口商品结构和出口市场结构，提高出口规模和效益。

大力发展非公有制经济。认真落实鼓励支持非公有制经济发展的政策措施，强化政府引导作用，为非公有制经济发展创造良好的政策环境、舆论环境、市场环境、金融环境和法制环境。进一步加强对中小企业的信息服务、金融服务、人才服务和技术服务，尤其要健全信用担保体系，鼓励和支持现有的信用担保机构，扩大信用规模，提高服务水平。实施"中小企业成长工程"，重点扶持一批技术含量高、产品附加值高、特色鲜明的项目，发展一批劳动密集型、资源综合利用型和科技型的企业。

（八）加强精神文明和民主法制建设，为社会经济发展提供保障

以建设社会主义核心价值体系为根本，围绕社会事业全面发展的目标，进一步加强精神文明和民主法制建设，创新社会管理体制，为夺取全面建设小康社会新胜利提供保证。

开展群众性创建活动。坚持以邓小平理论和"三个““代表””"重要思想为指导，广泛开展群众性精神文明创建活动，进一步弘扬"包容诚信、务实创新"的西宁精神，在市民中倡导学习意识、公德意识、诚信意识、包容意识和科学、文明、健康、和谐的生活方式，把思想教育与整治环境、德治人心与法治社会有机结合起来，加强社会公德、职业道德、家庭美德、个人品德建设，培养和塑造有理想、有道德、有文化、有纪律的文明市民，努力提高城乡文明程度。全面贯彻党的民族宗教政策，推进民族团结进步事业。进一步加强"双拥"工作，巩固军政军民团结。

推进民主法制建设。按照建设法治型政府的要求，自觉地接受人大及其常委会法律监督和政协的民主监督，认真办理人大““代表””、政协委员的意见、建议和提案。加强基层民主政治建设，建立健全各项民主管理制度。认真实施"五五"普法规划，推进"依法治市"工作，深入开展法制宣传教育和法治实践活动，提高公民的法律意识和政府的依法行政水平。

强化社会管理。不断完善社会治安防控体系，强化公共安全管理，提高化解矛盾、处置突发事件能力，严厉打击各种犯罪活动，保障人民生命财产安全。加强安全生产管理，落实安全生产责任制，坚决消除事故隐患，严防重特大事故发生。不断健全分类管理、分级负责、条块结合、属地为主的应急管理体制，有效应对自然灾害、事故灾难、公共卫生及社会安全等事件，提高危机管理和抗风险能力。认真做好人民来信来访工作，积极排查矛盾，化解纠纷，坚持依法拆迁、"阳光"拆迁，妥善解决征地、拆迁、企业改制等过程中出现的热点难点问题，全力维护社会和谐稳定。

我们将高度重视物价问题，采取有力措施，通过支持发展生产，完善储备体系建设，加强粮油、肉食、蔬菜等主要商品的生产、供应和市场调控，确保基本生活必需品供应不断档。健全产品供求和价格应急预案，进一步加强市场监管，抑制价格总水平过快上涨。深入开展食品卫生、产品质量等整治活动，让人民群众吃上安全食品、用上放心产品。

三、加强政府自身建设，推进各项工作全面落实

面对新的形势、新的任务，我们将牢记全心全意为人民服务的宗旨，决不辜负全市各族人民的期望和重托，切实加强自身建设，不断提高工作能力和服务水平，扎实做好各项工作，全面完成本次会议确定的各项任务，努力做到为人民服务、对人民负责、受人民监督、让人民满意。

解放思想，勇于创新。面对新形势，解决新问题，我们必须进一步解放思想，牢牢抓住发展这个第一要务不放松，站在全局的高度认识问题、分析问题，始终保持昂扬向上、奋发有为的精神状态，抢抓机遇，开拓创新，努力消除阻碍发展的思想瓶颈和体制障碍，坚决革除影响发展的传统观念和意识，不断创新思路，勇于破解工作中遇到的难题，坚持以新思维、新举措推动工作，力求工作有起色、有亮点，不断实现工作上的新突破。

转变职能，提高效率。围绕深入贯彻落实科学发展观，进一步开展学习型、服务型、诚信型、和谐型机关和学习型班子、学习型干部创建活动，强化公务员综合素质培训。注重研究经济社会发展中的重大问题，进一步增强政府工作的系统性和前瞻性。严格执行行政法律法规，规范行政程序，提高依法行政水平。进一步抓好绩效管理，强化效能监察，提高行政效率。加强政府公共服务职能和社会管理职能，努力提供更多的公共产品和公共服务。

改进作风，狠抓落实。弘扬求真务实的精神，大兴调查研究之风，始终把政府工作放在为民发展、为民致富、为民谋利上，讲实话，办实事，求实效，不搞形式主义，不做表面文章，坚持真抓实干，一抓到底。加强环境建设，简化办事程序，提高工作效率，坚决消除推诿扯皮现象，严肃查处不作为、乱作为行为。严格落实工作责任制，进一步强化目标责任考核，加强对重点工作、重点项目的督促检查，加强各地区、各部门之间的配合，在全市上下形成谋发展、抓落实的浓厚氛围。

艰苦奋斗，廉洁从政。牢固树立正确的权力观、地位观和政绩观，大力弘扬谦虚谨慎、艰苦奋斗的工作作风，认真落实党风廉政建设责任制，健全教育、制度、监督并重的惩治和预防腐败体系。加强对建设工程招投标、经营性土地招拍挂、产权交易、资源配置、政府采购等重点领域的管理和监督，严肃查处违法违纪行为。加强对公务员的教育、管理，进一步树立勤政廉政新形象。

各位代表，今年是推进"十一五"发展的关键之年，任务繁重，使命光荣。我们要在省委、省政府和市委的正确领导下，深入落实科学发展观，以"人一之，我十之"的精神，开拓进取，奋发有为，把西宁建设成更加繁荣、更加美丽、更加宜居的青藏高原现代化中心城市，为建设富裕文明和谐新青海做出新的更大的贡献！
